# The Cyanotoxin BMAA Induces Heterocyst Specific Gene Expression in *Anabaena* sp. PCC 7120 under Repressive Conditions

**DOI:** 10.3390/toxins10110478

**Published:** 2018-11-16

**Authors:** Alexandra A. Popova, Tatiana A. Semashko, Natalia V. Kostina, Ulla Rasmussen, Vadim M. Govorun, Olga A. Koksharova

**Affiliations:** 1 Institute of Molecular Genetics, Russian Academy of Sciences, Kurchatov Square, 2, 123182 Moscow, Russia; alexandra.a.popova@gmail.com; 2 Winogradsky Institute of Microbiology, Research Center of Biotechnology, Russian Academy of Sciences, Prospekt 60 let Oktyabrya, 7/2, 117312 Moscow, Russia; 3 Scientific-Research Institute of Physical-Chemical Medicine, 119435 Moscow, Russia; t.semashko@gmail.com (T.A.S.); govorun@hotmail.ru (V.M.G.); 4 Soil Science Faculty, Lomonosov Moscow State University, Leninskie Gory, 1-12, 119991 Moscow, Russia; nvkostina@mail.ru; 5 Department of Ecology, Environment and Plant Sciences, Stockholm University, 106 91 Stockholm, Sweden; ulla.rasmussen@su.se; 6 Belozersky Institute of Physical-Chemical Biology, Lomonosov Moscow State University, Leninskie Gory, 1, 40, 119992 Moscow, Russia

**Keywords:** BMAA, cyanobacteria, cyanotoxin, heterocyst differentiation, gene expression

## Abstract

Cyanobacteria synthesize neurotoxic β-*N*-methylamino-l-alanine (BMAA). The roles of this non-protein amino acid in cyanobacterial cells are insufficiently studied. During diazotrophic growth, filamentous cyanobacteria form single differentiated cells, called heterocysts, which are separated by approximately 12–15 vegetative cells. When combined nitrogen is available, heterocyst formation is blocked and cyanobacterial filaments contain only vegetative cells. In the present study, we discovered that exogenous BMAA induces the process of heterocyst formation in filamentous cyanobacteria under nitrogen-replete conditions that normally repress cell differentiation. BMAA treated cyanobacteria form heterocyst-like dark non-fluorescent non-functional cells. It was found that glutamate eliminates the BMAA mediated derepression. Quantitative polymerase chain reaction (qPCR) permitted to detect the BMAA impact on the transcriptional activity of several genes that are implicated in nitrogen assimilation and heterocyst formation in *Anabaena* sp. PCC 7120. We demonstrated that the expression of several essential genes increases in the BMAA presence under repressive conditions.

## 1. Introduction

β-*N*-methylamino-l-alanine (BMAA) is synthesized by many strains of cyanobacteria [1,2,3,4,5]. Biomagnifications of BMAA via food chains lead to toxin accumulation in brain tissues and neurodegeneration [6,7,8]. Phytoplankton BMAA synthesis [3,9,10,11,12] represents a potential threat to human health [7,13]. Thus, there is no doubt about the topicality of investigations of this cyanotoxin synthesis and its functional significance within producers’ cells. Nevertheless, modern understanding of the regulation of BMAA synthesis and its functions in cyanobacteria is still not completed [14,15]. It is known that unicellular non-dizotrophic strains of *Microcystis* and *Synechocystis* synthesize BMAA in the course of nitrogen starvation, while the addition of ammonium or nitrate abolishes this synthesis [12,16]. Furthermore, it has been shown that BMAA may participate in regulation of chlorosis under nitrogen deprivation in cyanobacteria *Synechocystis* sp. PCC 6803 [17].

Currently there are only two published studies on the impact of BMAA on diazotrophic cyanobacteria. It was found out that that exogenous BMAA decreases the enzyme activity of nitrogenase in the diazotrophic strain *Anabaena (Nostoc*) sp. strain PCC 7120 [18,19]. Moreover, we have discovered that this cyanotoxin inhibits heterocyst formation in filamentous cyanobacteria under nitrogen deprivation [19]. To learn the reason behind the inhibition of heterocyst formation we applied quantitative polymerase chain reaction (qPCR) analysis and tested the transcriptional activity of several genes that control cell differentiation in *Anabaena* sp. PCC 7120 during nitrogen step-down. We discovered that addition of exogenous BMAA leads to down-regulation of *hetR* and *hepA* genes in *Anabaena* sp. PCC 7120 [19]. Both of these genes are essential for the differentiation of heterocysts [20]. BMAA also inhibits expression of gene *nifH* and nitrogenase enzyme activity in cyanobacteria, which already possessed mature heterocysts before BMAA treatment [19]. Based on our data, the results obtained by Downing et al. [16] can be considered from a new point of view. We can hypothesize that the enhancement of BMAA synthesis by non-diazotrophic cyanobacteria during nitrogen starvation could be an allelopathic tool against nitrogen-fixing cyanobacteria that is used to scavenge their released resources, including nitrogen. One can assume that BMAA synthesis may be used as a strategy in antagonistic interactions between different cyanobacterial species. Moreover, perhaps other representatives of marine phytoplankton, such as diatoms and dinoflagellates, that are capable of synthesizing BMAA [1,10,11], could also use BMAA for competitive advantages. Of course, this assumption needs experimental verification.

However, the impact of BMAA on diazotrophic cyanobacteria in nitrogen-replete conditions remains unknown. In this study, we investigated exogenous BMAA effect on the *Anabaena* sp. PCC 7120 in the presence of nitrogen. We report new unexpected BMAA regulatory effect on the *Anabaena* sp. PCC 7120 (hereafter named *Anabaena* 7120). Cyanotoxin BMAA induces heterocyst specific gene expression in the cyanobacterium under repressive conditions.

## 2. Results and Discussion

### 2.1. β-N-Methylamino-l-Alanine (BMAA) Induces Formation of Non-Functional Heterocyst-Like Cells in Anabaena 7120 in Nitrogen-Replete Conditions

**Figure 1:**
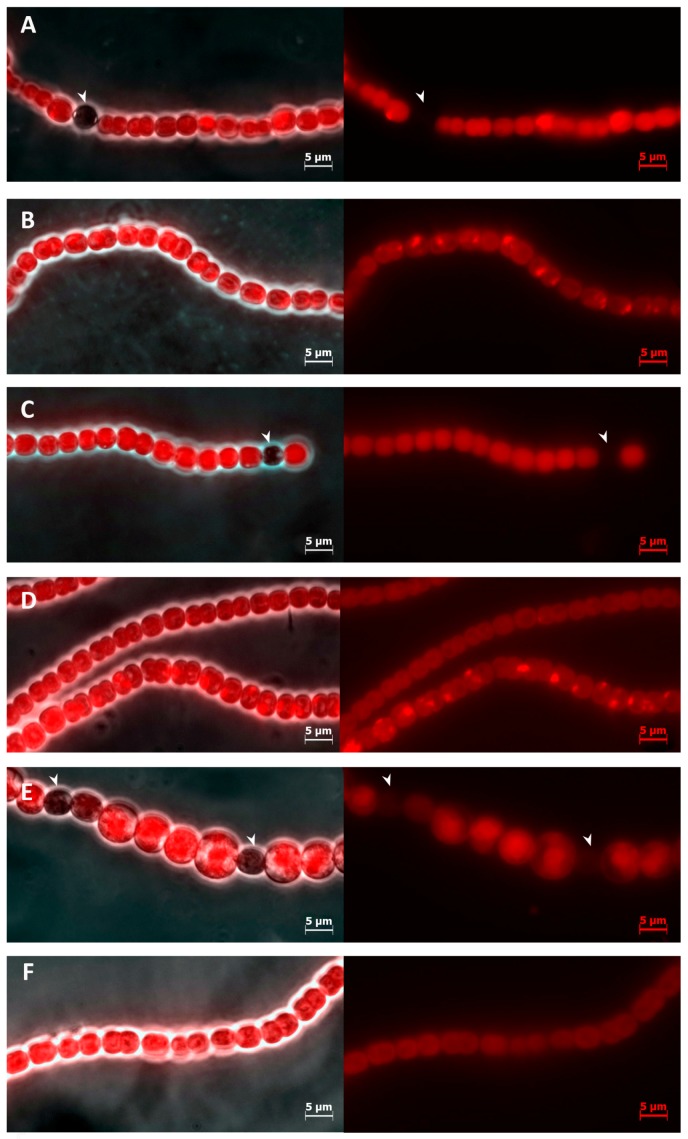
Filaments of *Anabaena* 7120 on different nitrogen sources after 72 h of cultivation are shown. (**A**) *Anabaena* 7120 grown on BG11_0_; (**B**) *Anabaena* 7120 grown on BG11_N_ with 17 mM sodium nitrate; (**C**) *Anabaena* 7120 grown on BG11_N_ with 17 mM sodium nitrate and 20 µM BMAA; (**D**) *Anabaena* 7120 grown on BG11_N_ with 5 mM ammonium chloride; (**E**) *Anabaena* 7120 grown on BG11_N_ with 5 mM ammonium chloride and 100 µM BMAA; (**F**) cyanobacteria after 72 h of incubation with 20 µM BMAA and 250 µM glutamate on nitrate-containing medium. On the left panels, cyanobacterial filaments are shown as a combination of light field and fluorescent images. The right panels show autofluorescence of chlorophyll. Heterocyst-like cells do not show fluorescence (arrows).

*Anabaena* 7120 forms filaments that consist of solely vegetative cells in the combined nitrogen presence (Figure 1B,D). In spite of this, BMAA addition caused the appearance of round-shaped dark cells after 48 h of incubation and more of them could be found later (after 72 h) (Figure 1C,E). These cells did not exhibit autofluorescence of chlorophyll as do normally mature heterocysts (Figure 1A) [21,22,23]. In addition, rudiments of the polar bodies could be noticeable at 100-fold magnification in some cells (Figure 1C). Wherein, these heterocyst-like cells were smaller, or comparable in size to vegetative cells (Figure 1C,E), while mature heterocysts in *Anabaena* 7120 are usually larger than vegetative cells [24]. In addition, we previously observed that the sizes of vegetative cells slightly increase in *Anabaena* 7120 grown in diazotrophic conditions in the presence of BMAA [19]. The same effect of BMAA was detected in nitrogen-containing medium where the average sizes of vegetative cells were determined as 11.50 ± 1.78 µm^2^ and 15.27 ± 3.23 µm^2^ in the untreated and BMAA-treated samples, respectively. The mean sizes of heterocyst-like cells were 11.13 ± 3.85 µm^2^, (*p* < 0.05).

We estimated the presence and frequency of heterocysts and heterocyst-like cells in *Anabaena* 7120 cultures grown for 72 h in three different conditions: (1) in nitrate-containing medium (BG11_N_) (only vegetative cells present in the culture) (2) in nitrate-containing medium (BG11_N_) with 20 µM BMAA added (heterocyst-like cells present in the culture); (3) in nitrogen-free medium (BG11_0_) (mature functional heterocysts present) (Figure 1, Table 1).

**Figure 2:**
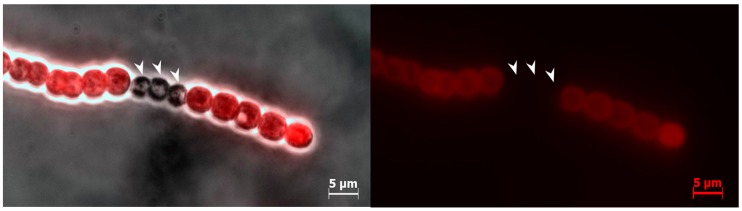
*Anabaena* 7120 filaments with multiple heterocyst-like cells after 72 h of cultivation on nitrate with BMAA (20 µM) are presented. On the left panel, cyanobacterial filament is shown as a combination of light field and fluorescent images. The right panel shows autofluorescence of chlorophyll. Heterocyst-like cells are nonfluorescent (arrows).

**Figure 3:**
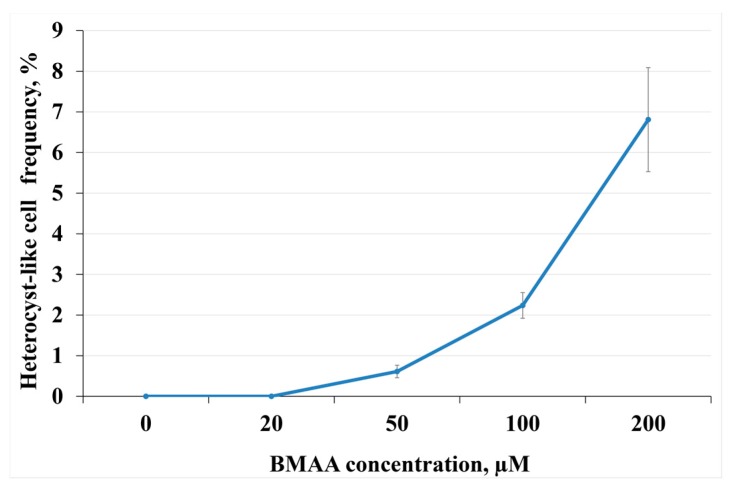
Dependence of heterocyst-like cells frequency on BMAA concentrations is shown. *Anabaena* 7120 cells were exposed with BMAA for 72 h in the medium containing ammonium (5 mM) (as nitrogen source).

The frequency of heterocyst-like cells was approximately 3.73% ± 1.74% after 72 h of incubation in liquid medium containing nitrate and 20 µM BMAA that was significantly different from the untreated nitrate-grown cells (Table 1). Doubled and multiple adjacent heterocyst-like cells were also observed in the presence of BMAA (Figure 2). Note, that filaments of *Anabaena* 7120 grown with nitrate survived only at 20 µM BMAA, while cyanobacterial filaments grown in medium with ammonium could stand concentrations of BMAA up to 200 µM. Furthermore, with the increase of BMAA concentration, the heterocyst-like cell frequency also increased (Figure 3).

**Figure 4:**
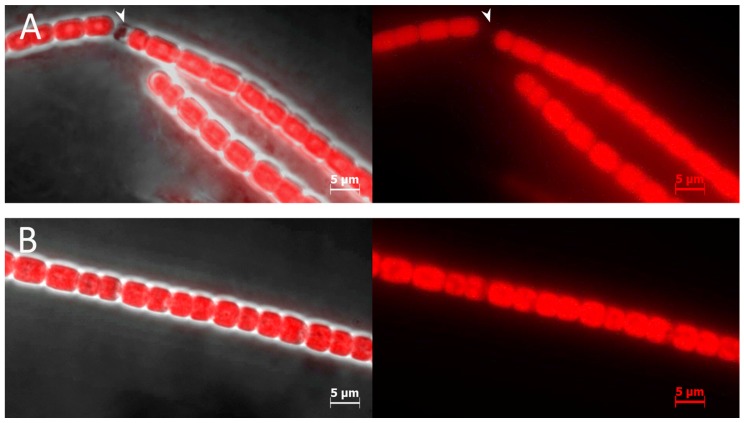
Filaments of *Nostoc* sp. strain 8963 after 72 h of cultivation on nitrate-medium are presented. (**A**) *Nostoc* sp. strain 8963 with 100 µM BMAA treatment; (**B**) *Nostoc* sp. strain 8963 without BMAA treatment. On the left panels, cyanobacterial filaments are shown as a combination of light field and fluorescent images. The right panels show autofluorescence of chlorophyll. Heterocyst-like cell does not show fluorescence (arrows).

BMAA induces heterocyst-like cells formation under nitrogen-replete conditions in symbiotic diazotrophic cyanobacteria: *Nostoc* sp. strain 8963 (Figure 4) and *Nostoc punctiforme* PCC 73102 (data not shown). As we found previously, these *Nostoc* strains could be exposed to higher BMAA concentrations than *Anabaena* 7120 [19], so we treated the *Nostoc* strains with 100 µM BMAA (Figure 4).

The derepression effect of BMAA is eliminated by the addition of glutamate (Figure 1F). This observation is consistent with the recent results [19], which have demonstrated that glutamate eliminates repressive activity of BMAA on cell differentiation under nitrogen starvation. This agrees with a consideration that BMAA and its carbamate are glutamate receptor agonists [25].

To determine the functionality of heterocyst-like cells we used the acetylene reduction assay (ARA) [26] to measure nitrogenase activity in *Anabaena* 7120 that possesses heterocyst-like cells, which have appeared after BMAA treatment of culture in the growth medium BG11_N_ (Table 1). As control samples two cultures of *Anabaena* 7120 incubated in medium with nitrate (BG11_N_) and in medium without nitrogen (BG11_0_) (Table 1) were used. There was no nitrogen fixation activity in the cyanobacterial culture containing heterocyst-like cells (BG11_N_, 20 µM BMAA) (Table 1). Thus, it can be concluded that the heterocyst-like cells are not functionally heterocysts.

### 2.2. Comparison of BMMA and Other Non-Proteinogenic Amino Acids Effects on Heterocyst Derepression

The effect of BMAA on nitrogen-grown *Anabaena* 7120 had similarities and differences in comparison with other non-proteinogenic amino acids that are well-known to induce derepression of heterocyst differentiation. Among them are L-methionine sulfoximine (MSX), 7-azatryptophan (azaTrp) and beta-2-thienylalanine (B2TA) [27,28,29,30]). Both BMAA and MSX significantly inhibit cyanobacterial growth under nitrogen-replete conditions [28]. It was found that MSX inhibits the activity of the enzyme glutamine synthetase (GS) and thus MSX derepresses mature heterocyst formation in the presence of nitrogen [27,28]. AzaTrp and B2TA do not inhibit GS activity, but azaTrp suppresses the activity of glutamine oxoglutarate aminotransferase (GOGAT), that leads to the accumulation of 2-oxoglutarate (2-OG) in the medium, which participates in signaling a response to nitrogen deprivation and in formation of heterocysts [27,28,29]. However, in contrast to BMAA and B2TA, the non-proteinogenic amino acids MSX and azaTrp induce formation of functional heterocysts [27,28].

So far, information about the mechanisms of non-proteinogenic amino acids effects on heterocyst formation is scarce. From our previous research, we know that BMAA suppresses genes involved in heterocyst formation and functionality under conditions of nitrogen deprivation [19]. In this study we found that heterocyst-like cells are formed in cyanobacteria due to the presence of BMAA in a nitrogen-rich medium, so we asked a question: does BMAA presence change the specific heterocyst gene expression in the nitrogen-grown cells of *Anabaena* 7120? To answer this question quantitative PCR was applied.

### 2.3. BMAA Induces Heterocyst-Specific Gene Expression in Anabaena 7120 under Repressive Conditions

Heterocysts of cyanobacteria are highly specialized cells for the process of nitrogen fixation. During nitrogen deprivation, cell differentiation is initiated by nitrogen deficiency in the growth medium. Cyanobacterial cell differentiation is an energy-consuming and finely-regulated, complex process [20,31,32]. Therefore, heterocyst formation normally is repressed in cyanobacteria in the nitrogen-replete growth medium [33] (Figure 1B,D). Since the addition of BMAA leads to the formation of heterocyst-like cells in *Anabaena* 7120 under repressive conditions, we used reverse transcription quantitative PCR (RT-qPCR) (Table 2) to estimate the transcription activity levels of several genes, involved in heterocyst formation and nitrogen assimilation. We discovered that the expression of heterocyst-specific genes (*hetR*, *hepA*, *nifH*) and genes participating in nitrogen metabolism (*glnA* and *gltS*) significantly increased compared to the control after 48 h of incubation in the presence of 20 µM BMAA in the BG11_N_ medium with sodium nitrate (Table 2). Note, the dark non-fluorescent heterocyst-like cells could be found also after 48 h of cyanobacterial growth in the presence of BMAA.

Protein HetR interacts with the promoter of the *hepA* gene and regulates its expression during nitrogen step-down [34]. In the absence of BMAA, the *hetR* gene was not expressing in *Anabaena* 7120 (Table 2). Surprisingly, under BMAA treatment, the *hetR* gene expression was significantly upregulated within 48 h (Table 2). In a study by Buikema and Haselkorn [35] the presence of heterocyst-liked cells was reported in a wild type of *Anabaena* 7120 carrying a plasmid containing the *hetR* region, encoding HetR protein, when grown in the presence of nitrate. As in our study, the culture with heterocyst-like cells did not show any nitrogen fixation activity according to acetylene reduction assay (ARA) [35]. Under nitrogen depleted growth conditions the *hepA* expression follows after the *hetR* expression in proheterocysts [20,32,36]. In the control sample, as expected, the expression of both genes, *hepA* and *hetR*, was repressed (Table 2). In contrast, in the presence of BMAA, *hepA* gene was upregulated already after four hours of BMAA treatment (Table 2). The products of the *hetR* and *hepA* genes are required for heterocyst formation; thus, unpredictable induction of the transcription of these genes could be an explanation as to why a cyanobacterium forms heterocyst-like cells in the presence of BMAA (Figure 1C).

The *nifHDK* operon transcription is required at the final stages of heterocyst maturation, when subunits of nitrogenase enzyme have to be synthesized [37]. We found that *nifH* gene expression under BMAA treatment was upregulated in repressive conditions in comparison with the control sample (Table 2). However, such unexpected *nifH* gene expression in the presence of both nitrate and BMAA did not provide conditions for active nitrogenase enzyme synthesis, since the heterocyst-like cells did not show any of the acetylene reduction activity (Table 1).

The *ntcA* gene encodes a global regulatory protein of nitrogen metabolism, a transcription factor NtcA [38]. BMAA did not significantly affect the expression of this gene during the first 24 h compared to control sample. Its expression increased slightly after 48 h of incubation with BMAA.

Thus, in the presence of BMAA the cell control of key genes’ expression unexpectedly changed. The genes, involved in the heterocyst and nitrogenase formation, are supposed to be silent under nitrogen-replete conditions; however, they are activated, what leads to heterocyst-like non-functional cells formation.

### 2.4. BMAA Induces glnA and gltS Gene Expression in Anabaena 7120 under Repressive Conditions

We examined the BMAA action also on the expression of three genes (*glnA*, *gltS* and *nirA*), whose products are important enzymes of cyanobacteria nitrogen metabolism (Table 2). The *glnA* and *gltS* genes encoded glutamine synthetase (GS) and glutamate synthase (GOGAT), respectively. These enzymes are key players in nitrogen assimilation [39,40,41]. In our experiments, the expression of the *glnA* and *gltS* genes increased significantly after 48 h in the BMAA-treated sample during growth in a nitrate-containing medium. In addition, the transcript levels of the *nirA* gene did not noticeably change after the BMAA treatment (Table 2). The product of the *nirA* gene is the nitrite reductase, one of the main enzymes implicated in nitrate assimilation [42,43]. *Anabaena* 7120 was more resistant to higher concentrations of BMAA in the ammonium-containing medium than in the medium with nitrate (Figure 3). Thus, it could be proposed that the possible BMAA target could be related to the functionality of enzymes involved in nitrate assimilation or to expression of *narB* and *nirA* genes encoding nitrate and nitrite reductase, respectively [44]. However, our results did not support this statement, at least not with respect to *nirA* gene expression (Table 2).

The unexpected expression of heterocyst specific genes in repressive conditions indicates a false nitrogen depletion signal in cyanobacterial cells. What kind of the nitrogen limitation signal could arise in the cyanobacterial cells in the presence of BMAA in the nitrogen-containing growth medium? It is known that the 2-OG is involved in the signaling response to nitrogen stress [31,32,45]. Moreover, frequency of heterocyst rises with the increase of 2-OG concentration in the growth medium [45]. It is also known that 2-OG is involved and metabolized via the GS/GOGAT pathway [46]. We can hypothesize that BMAA, as an analogue of glutamate [25,47], can compete with glutamate for binding to the GS enzyme. Therefore, GS/GOGAT cycle activity could be impaired by the addition of BMAA. Recently researchers found that this cyanotoxin affects the metabolism of nitrogen in diatoms via the GS/GOGAT pathway, possibly through the inhibition of ammonium assimilation [48]. Thus, the disturbance of the GS/GOGAT pathway by BMAA could lead to accumulation of the signal molecule 2-OG. In its turn, 2-OG stimulates the DNA-binding activity of nitrogen global regulator NtcA [40,49] and triggers the expression of the genes involved in heterocyst formation in restrictive conditions. Recently it was reported that BMAA disturbs metabolism of amino acids (alanine, aspartate and glutamate) in eukaryotic cells [50,51]. Hence, if BMAA interferes in these pathways in cyanobacteria, it could affect nitrogen control that causes improper regulation of gene expression. Verification of this hypothesis requires further exciting studies.

Summarizing, we can conclude that BMAA influences nitrogen metabolism and gene expression of filamentous nitrogen-fixing cyanobacteria, which leads to disturbances in their development. In particular, under conditions of nitrogen starvation BMAA suppresses the formation of heterocysts, and under conditions of nitrogen excess it induces a signal of nitrogen deficiency in some way. This, in turn, leads to the expression of genes that are usually silent under nitrogen-replete conditions and to the formation of heterocyst-like cells. New experiments with the application of mutagenesis and biochemistry methods can help us to understand the details and clarify the role of BMAA in the metabolism of cyanobacteria.

From an ecological point of view, future experiments on the joint cultivation (mixed cultures) of different cyanobacteria species, or cyanobacteria and diatoms, followed by metabolites monitoring, are great of interest. Cyanobacteria and algae are in complex relationships, including symbiosis and competition [52,53,54,55,56,57]. Allelopathic functions of other cyanobacterial toxin, microcystin, have been investigated in several studies [58,59,60,61,62,63]. It would be interesting to find out the possible role of BMAA in allelopathy.

## 3. Conclusions

We found an unusual regulatory effect of BMAA that induced the transcription of several essential genes implicated in heterocyst formation, nitrogen fixation and nitrogen assimilation in *Anabaena* sp. PCC 7120. This BMAA regulatory effect leads to the formation of non-functional dark non-fluorescent heterocyst-like cells under repressive conditions. The induction of heterocyst-like cells was also observed in two different *Nostoc* strains. The regulatory effect of BMAA could be canceled by glutamate addition. Data obtained are important for further fundamental studies of the regulatory role of cyanobacterial secondary metabolites.

## 4. Materials and Methods

### 4.1. Cyanobacterial Strain and Cultivation Conditions

Cyanobacterium *Anabaena* sp. PCC 7120 (in short *Anabaena* 7120) was received from the Pasteur Culture collection of Cyanobacteria, Paris, France.

*Nostoc* sp. strain 8963 and *Nostoc punctiforme* PCC 73102 (ATCC 29133) are symbiotic isolates from, respectively, *Gunnera prorepens* [1] and cycad *Macrozamia* sp. [64].

Filamentous nitrogen-fixing cyanobacteria *Anabaena* 7120, *Nostoc punctiforme* PCC 73102, *Nostoc* sp. strain 8963 were grown in 100 mL Erlenmeyer flasks containing 25 mL of BG11 liquid medium [64]. We used nitrogen-free medium (BG11_0_) or medium (BG11_N_) containing 17.6 mM sodium nitrate or 5 mM ammonium chloride. Cultures were grown at 25 °C on a shaker at 63 rpm and with continuous shaking at a light intensity of 18 µmol photons m^−2^s^−1^.

### 4.2. Chlorophyll A Measurements

Concentration of Chl*a* was measured as described in [19]. Samples were collected during six days of *Anabaena* 7120 growth with BMAA. Cells in the aliquots of cyanobacterial suspensions were degraded by centrifugation, followed by vortexing in 90% ethanol for Chl *a* release into supernatant. Measurements were conducted spectrophotometrically at 665 nm and 750 nm using Ultrospec 3000 (Pharmacia Biotech, Cambridge, UK). Concentration of Chl*a* was calculated according to [65].

### 4.3. Nitrogenase Activity Measurements

The nitrogenase activity was measured using ARA combined with gas chromatography according [8,19] with some differences described below and calculated as amount of acetylene (C_2_H_2_) reduced to ethylene (nmol C_2_H_4_ µg Chl a^−1^h^−1^) produced according to Capone and Montoya [26]. We collected three aliquots of cell suspension from the each flask into glass vials to provide three technical replicates; 1 mL of the gas phase from each vial was analyzed for ethylene content using gas chromatography (Kristall 2000, RPC «Meta-Chrom», Russia, Mari El Republic, Yoshkar-Ola) with a flame ionization detector (GC-FID). The amount of ethylene was measured with the following device parameters: column length 1 m, diameter 3 mm, filler Porapak N 80/100 mesh (Sigma-Aldrich, Darmstadt, Germany), column temperature +60 °C, detector temperature +160 °C, evaporator temperature +100 °C, flow rate of carrier gas (N_2_) 50 mL min^−1^, air—280 mL min^−1^, hydrogen—28 mL min^−1^). Peaks were displayed with the software NetChrom V 2.1 (RPC «Meta-Chrom», Russia, Mari El Republic, Yoshkar-Ola). Concentrations of Chl*a* were used for nitrogenase activity normalization. All experiments were performed in five biological repeats.

### 4.4. Fluorescence Microscopy

The control and BMAA treated cells of cyanobacteria were investigated microscopically after 48, 72 and 96 h after 20 μM BMAA additions. The observations were performed by using a fluorescence microscope Axiovert 200M and Plan-Neofluar (Carl Zeiss GmbH, Jena, Germany). Measurements were made using the Microscope Software AxioVision 4.5. Heterocyst-like cells’ frequency was defined as the percentage of heterocyst-like cells of the total cyanobacterial cell population. This was determined by counting the heterocyst-like cells and vegetative cells numbers along the filaments. The total number of cells counted was no less than 650 cells per sample.

### 4.5. RNA Extraction and Reverse Transcription Quantitative PCR (RT-qPCR)

Total RNA isolation from *Anabaena* 7120 was performed by using the Trizol (Invitrogen, CA, USA) reagent and cDNA synthesis was conducted by H-minus Mu-MLV reverse transcriptase (200 U/µL) (Thermo Scientific, #EP0451, Waltham, MA, USA) treatment as described in [19]. To quantify expression levels of target genes (Table 2), RT-qPCR was performed as described in [19]. The gene-specific primers (Table 3) [19] were synthesized at the Syntol Company (Moscow, Russia). SYBR Green I dye (Invitrogen) was used for amplicon staining. The comparative −2^ΔΔ*C*t^ method was used to calculate and analyze the relative gene expression [66]. All Ct values were normalized to *rnpB* [67].

### 4.6. Statistical Analysis

Three or five biological replicates were performed and the average with standard deviation was calculated for all data. Three or four technical repeats were performed within each measurement. The Student’s *t*-test was used to determine the statistical significance determination. The significance of mRNA level change was identified by using a *t*-test with multiple and Benjamini–Hochberg adjustment for *p*-values [68]. One-way analysis of variance (ANOVA) with the Benjamini–Hochberg correction was applied for time groups’ comparison. Pairwise comparison was made using Tukey’s test. The R statistical software program was applied for the analyses.

## Figures and Tables

**Table 1:** The effect of BMAA on the frequency of heterocyst and heterocyst-like cells and on nitrogenase activity of *Anabaena* 7120.

	Growth Condition	^1^ Frequency of Heterocyst or Heterocyst-Like Cells, ^1^ %	^2^ Nitrogenase Activity, ^2^ nmol (Eth)/μg(Chl) h
1	BG11_N_	0.0	0.0
2	BG11_N_, 20 μM BMAA	3.73 ± 1.73	0.0
3	BG11_0_	5.14 ± 1.28	22.4 ± 3.84

^1^ Heterocysts and heterocyst-like cells frequency expressed in % of a total number of heterocysts and vegetative cells determined by fluorescence microscopy. ^2^ Nitrogenase activity was expressed as chlorophyll-normalized production of 1 nmol of ethylene per hour; Eth—ethylene, Chl—chlorophyll. The average values of three independent experiments for each experimental condition are presented. Frequency difference is significant for the condition (2) and the difference in nitrogenase activity is significant for the condition (3) in accordance with independent *t*-test (*p* < 0.05).

**Table 2:** Reverse transcription quantitative polymerase chain reaction (RT-qPCR) analysis of the nitrogen-regulated gene expression in *Anabaena* 7120 in the absence (control) or in the presence of BMAA (20 μM) after 4, 8, 24, 48 and 96 h of BMAA treatment in nitrate-containing medium ^1^.

Gene	Product		Relative Ratio in log_2_				
			4 h	8 h	24 h	48 h	96 h
*hetR*	HetR, Heterocyst differentiation protein	Control	−2.33 ± 0.02	0.64 ± 0.23	−1.03 ± 0.19	**0.09 ± 0.07**	0.15 ± 0.06
		BMAA	−2.26 ± 0.32	0.45 ± 0.41	−1.29 ± 0.22	**3.35 ± 0.38**	0.36 ± 0.22
*hepA*	HepA, Heterocyst differentiation protein	Control	**−0.53 ± 0.06**	**0.66 ± 0.21**	**−0.06 ± 0.13**	**0.09 ± 0.07**	0.15 ± 0.06
		BMAA	**1.35 ± 0.05**	**3.87 ± 0.36**	**1.83 ± 0.23**	**3.35 ± 0.38**	0.36 ± 0.22
*ntcA*	NtcA, Nitrogen-responsive regulatory protein	Control	−2.01 ± 0.01	2.50 ± 0.60	−1.35 ± 0.11	−0.12 ± 0.09	−0.65 ± 0.27
		BMAA	−1.36 ± 0.67	2.30 ± 0.35	−0.53 ± 0.10	1.31 ± 0.27	0.88 ± 0.22
*nifH*	Nitrogenase subunit	Control	−0.39 ± 0.07	**1.37 ± 0.63**	**1.47 ± 0.68**	**0.30 ± 0.50**	3.18 ± 2.82
		BMAA	0.50 ± 0.08	**3.44 ± 0.11**	**2.62 ± 0.07**	**6.57 ± 1.43**	4.82 ± 0.58
*glnA*	Glutamine synthetase	Control	1.51 ± 0.56	−0.56 ± 0.05	2.22 ± 0.95	**0.19 ± 0.16**	−2.60 ± 0.17
		BMAA	1.80 ± 0.04	−0.67 ± 0.99	2.17 ± 0.67	**2.70 ± 0.15**	1.02 ± 0.11
*gltS*	Glutamine-oxoglutarate-aminotransferase	Control	−1.66 ± 0.58	1.23 ± 0.15	−1.14 ± 0.40	**−0.24 ± 0.03**	0.65 ± 0.17
		BMAA	−1.95 ± 0.39	0.67 ± 0.28	−0.13 ± 0.01	**2.17 ± 0.16**	2.17 ± 0.12
*nirA*	Nitrite reductase	Control	−0.65 ± 0.16	1.40 ± 0.16	−0.67 ± 0.26	−1.51 ± 0.16	−4.54 ± 0.07
		BMAA	−1.10 ± 0.28	1.63 ± 0.19	−0.90 ± 0.26	−1.18 ± 0.18	−2.87 ± 0.15

^1^ Fold changes in gene expression are reported as log_2_ values. Each sample was measured in triplicate, and the standard deviation is indicated by error bars. Values were normalized to the *rnpB* transcript level. The expression of the “housekeeping” gene *rnpB* was not affected by the action of BMAA (data not shown). Significant differences of transcript levels between control and treated samples are shown inbold (*p* < 0.05) and were tested by Student’s *t*-test.

**Table 3:** Primers used for RT-qPCR.

Primer	Sequence (5′→3′)	Reference
nifH-F	CTATGCCTATCCGTGAAGG	[19]
nifH-R	CCAAGTTCATGATTAACTCGTC	[19]
hetR-F	AGTTACCCAGCAATCTTCCC	[19]
hetR-R	ATAGAAGGGCATTCCCCAAG	[19]
ntcA-F	GAGCTTTTCCTCCTGTTGTC	[19]
ntcA-R	ACCTATCCGACTTGTTTCCT	[19]
glnA-F	GGTGATACAGCCTTCTTTGG	[19]
glnA-R	CTTGGAAAGAATCTGTGGGG	[19]
nirA-F	CCAACAAAGGAGAAGGCAAT	[19]
nirA-R	AGAAACCACCAACTAACACG	[19]
hepA-F	TTCGGGTGAACTCATTAATACG	[19]
hepA-R	TTCTCTGACTCGCTTATTCAG	[19]
gltS-F	TAGAACATCGGGGTGGTTGT	[19]
gltS-R	CTACTCGCCAGCCCAATAC	[19]
rnpB-F	ACTGATTTGAGGAAAGTCCG	[67]
rnpB-R	CTTTGCACCCTTACCAAGAG	[67]
